# Introductory to Discussion on Dental Nomenclature

**Published:** 1896-10

**Authors:** S. B. Brown

**Affiliations:** Fort Wayne, Ind.


### Introductory to Discussion on Dental Nomenclature.

BY DR. S. B. BROWN, FORT WAYNE, IND.

Read before the Indiana State Dental Society, Indianapolis, Ind., June 30, 1896.

Faultless Anglo-Saxon is rare, but the glaring errors of speech daily assailing our ears, coming from those who have assumed the obligation of sustaining the reasonable requirements of a learned profession, are humiliating, as the popular estimate of a profession is justly based on the evidence of culture of its individual members.

We are often attracted to those giving seeming indication of an interesting personality only to experience a sudden disappointment, when, for example, inquiring for the health of a mutual friend to be answered: "I have not *saw* him recently; when I *seen* him last he was as usual."

Correct language frequently has an advantage over education as a social passport.

It is admissible sometimes to yield points in adapting our language to the comprehension of patients, those illiterate, not, however, to the extent of copying one who directs attention to a tooth locating it as the "down backest." Nor to accept the language of the Irishman who called for extraction and rebuked further inspection of teeth in his lower jaw by exclaiming: "Faith, it is over-head."

Within the recollection of many such crude names as "job"

was generally applied to a case of construction of artificial teeth; "plug" for filling; "pulling" for extracting is still projected into dental language. It it a common error for members to speak of our association as a "convention." It is superfluous to prefix the adjective "free" when naming the margin of the gums.

It is our misfortune that some are willing, by inattention to correct speech, to be rated as a misplaced professional man, or what is worse, to thereby compromise our profession.

This introductory seemed irresistible in complying with the duty assigned me by our Executive Committee to open a discussion on the report of the American Dental Asssociation's Committee on Dental Nomenclature.

